# Comparison of ChIP-Seq Data and a Reference Motif Set for Human KRAB C2H2 Zinc Finger Proteins

**DOI:** 10.1534/g3.117.300296

**Published:** 2017-11-16

**Authors:** Marjan Barazandeh, Samuel A. Lambert, Mihai Albu, Timothy R. Hughes

**Affiliations:** * Terrence Donnelly Centre for Cellular and Biomolecular Research, University of Toronto, Ontario M5S 1A8, Canada; † Department of Molecular Genetics, University of Toronto, Ontario M5S 1A8, Canada; ‡ Canadian Institutes for Advanced Research, Toronto, Ontario M5G 1M1, Canada

**Keywords:** KRAB C2H2 zinc finger proteins, endogenous retroelements, DNA-binding motif, ChIP-seq, C2H2 recognition code

## Abstract

KRAB C2H2 zinc finger proteins (KZNFs) are the largest and most diverse family of human transcription factors, likely due to diversifying selection driven by novel endogenous retroelements (EREs), but the vast majority lack binding motifs or functional data. Two recent studies analyzed a majority of the human KZNFs using either ChIP-seq (60 proteins) or ChIP-exo (221 proteins) in the same cell type (HEK293). The ChIP-exo paper did not describe binding motifs, however. Thirty-nine proteins are represented in both studies, enabling the systematic comparison of the data sets presented here. Typically, only a minority of peaks overlap, but the two studies nonetheless display significant similarity in ERE binding for 32/39, and yield highly similar DNA binding motifs for 23 and related motifs for 34 (MoSBAT similarity score >0.5 and >0.2, respectively). Thus, there is overall (albeit imperfect) agreement between the two studies. For the 242 proteins represented in at least one study, we selected a highest-confidence motif for each protein, utilizing several motif-derivation approaches, and evaluating motifs within and across data sets. Peaks for the majority (158) are enriched (96% with AUC >0.6 predicting peak *vs.* nonpeak) for a motif that is supported by the C2H2 “recognition code,” consistent with intrinsic sequence specificity driving DNA binding in cells. An additional 63 yield motifs enriched in peaks, but not supported by the recognition code, which could reflect indirect binding. Altogether, these analyses validate both data sets, and provide a reference motif set with associated quality metrics.

The human genome encodes ∼350 KRAB C2H2 zinc finger proteins (KZNFs), which encode a Kruppel-Associated Box (KRAB) domain, which is best known for its repressor activity (Schultz *et al.* 2002), followed by a tandem array of C2H2 zinc finger (ZNF) domains (up to 40), which mediate sequence-specific DNA binding. The ZNFs each contact three or more bases, and typically bind in tandem with an offset of three bases. The DNA sequence motifs recognized by the array of ZNFs therefore often resemble concatenation of the base preferences for the individual ZNFs (Wolfe *et al.* 2000), which can be predicted to some degree on the basis of “specificity residues” at positions –1, 2, 3, and 6 of the DNA-contacting α helix (Najafabadi *et al.* 2015a). The modular fashion of DNA recognition by C2H2-ZF proteins apparently facilitates adaptation, with evidence for positive selection on the specificity residues of many KZNFs (Emerson and Thomas 2009), such that many encode a unique sequence specificity (Najafabadi *et al.* 2015a).

The best-characterized function of the KRAB domain is to recruit TRIM28 (aka KAP1), which represses transcription by subsequent recruitment of SETDB1, a histone H3 lysine 9 (H3K9) trimethylase (Schultz *et al.* 2002). TRIM28 is involved in silencing endogenous retroelements (ERE); this observation led to the now widely accepted theory that KZNFs evolve rapidly to silence EREs (Matsui *et al.* 2010; Rowe and Trono 2011). A KZNFs *vs.* EREs “arms race” model (Jacobs *et al.* 2014) provides a readily understood mechanism for the evolution of new KZNFs, but does not explain the retention of so many of them; presumably, they take on other host functions. The KRAB domains vary in primary sequence, with an average sequence identity of ∼40%, and KZNFs vary in their protein–protein interactions (Schmitges *et al.* 2016), suggesting that they may also vary in effector function.

Detailed study of the KZNFs requires knowledge of their intrinsic DNA binding preferences (*i.e.*, motifs), and their genomic binding sites. General characteristics of their motifs can be gleaned from their protein sequences, *e.g.*, that two proteins should bind distinct motifs, but current “recognition codes” are too error-prone to obtain high-confidence motifs (Najafabadi *et al.* 2015b). Furthermore, it remains difficult to determine which C2H2 domains in a large array are likely to bind DNA (Brayer and Segal 2008); KZNFs typically carry many more tandem ZNFs than needed to specify individual loci in the human genome (2–40; median 12), and known motifs for human ZNF proteins often correspond to only part of the ZNF array (Najafabadi *et al.* 2015a). An additional challenge with ZNF proteins is that they often fail to yield motifs from *in vitro* analyses such as Protein Binding Microarrays (Badis *et al.* 2009; Wang 2014) and HT-SELEX (Jolma *et al.* 2013), possibly due to misfolding, lack of obligate cofactors, or the fact that many KZNFs have long binding sites, which would be poorly represented among the sequences in these experiments. Systematic application of these approaches has consequently yielded motifs for very few of the KZNFs (Badis *et al.* 2009; Jolma *et al.* 2013, 2015; Weirauch *et al.* 2013). Other approaches to determining sequence specificity of C2H2 proteins have been described, including SMILE-seq (Isakova *et al.* 2017), B1H (Noyes *et al.* 2008), and analysis of selected sites by context-dependent models (Zhao *et al.* 2009), but, to our knowledge, none has been tested on a large number of human proteins.

ChIP-seq and related methods [*e.g.*, ChIP-exo (Imbeault *et al.* 2017)] represent an alternative to rapidly obtain motifs for C2H2 zinc finger proteins. ChIP-seq is a relatively challenging means to obtain high-confidence motifs for TFs, due to the fact that the number of binding sites is relatively small (in comparison to, *e.g.*, HT-SELEX), that the genome sequence is highly nonrandom (with many sequence biases and repeated sequences), and that proteins can associate with DNA indirectly, such that cofactor motifs are often obtained (Encode-Project-Consortium 2012; Mathelier and Wasserman 2013). For KZNF proteins, the fact that EREs are often bound represents an additional confounding variable, because motif-finding tools [*e.g.*, MEME (Bailey *et al.* 2009)] typically assume that different binding sites are drawn from independent random sequences, while EREs are related by common origin. To address these issues, we have previously described Recognition Code-Assisted Discovery of regulatory Elements (RCADE), a computational method that employs the zinc finger recognition code to predict primary binding motifs from ChIP-seq data specifically for C2H2-ZF proteins (Najafabadi *et al.* 2015a). We utilized RCADE in two large-scale analyses of human C2H2-ZFs proteins (using tagged, inducible heterologous expression constructs in HEK293 cells) to produce motifs for dozens of human C2H2-ZFs (Najafabadi *et al.* 2015b; Schmitges *et al.* 2016).

A recent study described a similar ChIP-exo analysis of 221 human KZNFs (Imbeault *et al.* 2017), also in HEK293 cells (but using different constructs). This study did not describe DNA binding motifs, however. Here, we applied our existing data analysis pipeline to the data from the Imbeault *et al.* (2017) study, and compared the results to our own, and also to HT-SELEX, SMILE-seq, and other motifs from the literature, where available. Overall, the comparison reveals that the two data sets usually produce comparable motifs, and also largely agree on the sets of EREs bound by the individual KZNFs. This agreement is obtained even though most of the individual peaks do not overlap, suggesting that both studies sample from a larger set of genomic target sites. We introduce a web portal that summarizes the results of our analyses (http://kznfmotifs.ccbr.utoronto.ca/), and produce a set of high-confidence motifs, each together with a confidence statistic that represents its ability to predict binding sites in living cells.

## Materials and Methods

### Reprocessing the ChIP-exo data

Primary and processed ChIP-seq and ChIP-exo data were obtained from GEO accession numbers GSE76496 (Schmitges *et al.* 2016), and GSE78099 (Imbeault *et al.* 2017). The Trono ChIP-exo data were reprocessed as previously described (Najafabadi *et al.* 2015b; Schmitges *et al.* 2016). Briefly, the ChIP-exo reads were trimmed to 50 nucleotides and mapped to the human genome (hg19; build GRCh37) using Bowtie 2 (Langmead and Salzberg 2012). We used the “–very-sensitive” preset option, which allows the retention of one alignment for the multi-mapped reads; otherwise many ERE instances cannot be detected. The multi-mapping may affect the reads containing EREs due to their similar sequences by aligning them to the genomic repetitive regions other than their origin, and could result in erroneous peak calling. However, as we and others have described before (Najafabadi *et al.* 2015b), this is unlikely to be a problem regarding the enrichment of EREs, since these reads typically map to another instance of the same ERE, and vice versa (Day *et al.* 2010).

MACS 1.4.2 was used for peak calling (Zhang *et al.* 2008), where the provided input DNA data set (a combination of randomly selected peaks from all ChIP-exo) was used as the control. The biological replicates were merged into single samples, retaining all the peaks from all replicates, and merging the peaks in a maximum of 50 bp distance from each other into a single peak. For further analyses, the top 500 peaks with the highest enrichment scores were picked.

### Motif analysis

Non-ERE peaks were filtered using RepeatMasker track of the UCSC Table Browser (Karolchik *et al.* 2004). We used RCADE (Najafabadi *et al.* 2015a) using default settings and MEME (Bailey *et al.* 2009) with CentriMo, on both all and non-ERE peaks, giving priority to those obtained by RCADE and in non-ERE peaks, as will be described in the text. For further detail, see Schmitges *et al.* (2016). Enrichment of motifs from ChIP data and other sources were tested by calculating AUROC for differentiating the top 500 ChIP peaks in a range of ±250 bp from the summit from dinucleotide shuffled sequences, using the single maximum PWM scoring match for each 501-base sequence as its score.

### Motif similarity analysis

Motif similarity was measured using the Motif Similarity Based on Affinity of Targets (MoSBAT) method (Lambert *et al.* 2016) using the energy scores option. To make similarity measurements consistent across datasets we set the sequence background to *n* = 100,000 and *l* = 100.

### ERE enrichment

The ERE enrichment was calculated as the proportion of the top 500 peaks overlapping any transposon and retroelement instances present in the RepeatMasker. The Pearson correlations (*r*) between the overlapped EREs of all possible Hughes and Trono pairs (matching and unmatching within the same set of 39) were calculated using R (v. 3.2.3).

### Data availability

The detailed results of our analyses for the 242 KZNFs are available at the web portal of the paper (http://kznfmotifs.ccbr.utoronto.ca/). For each KZNF, it contains motifs from different sources, a heatmap of the pairwise motif similarity scores between different motifs, the overlap between all peaks and top 500 peaks of the Hughes and Trono ChIP data, where available, and the ERE enrichment of the peaks.

## Results

**Figure 1:**
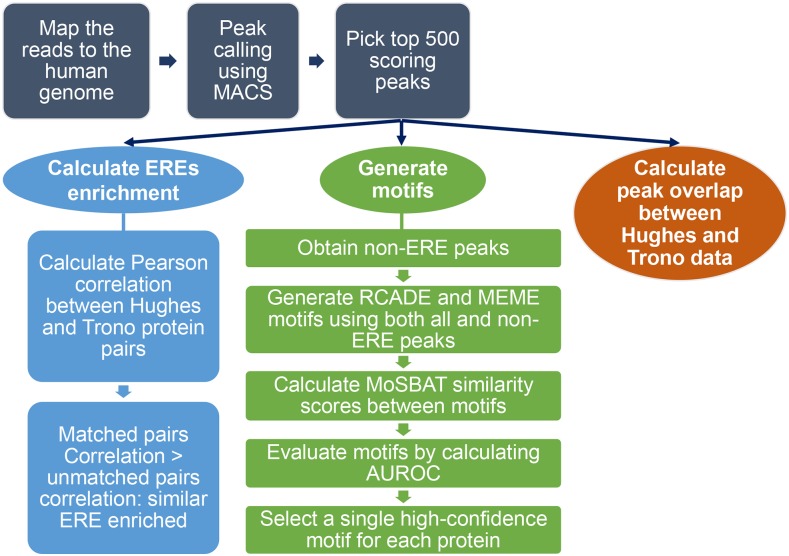
Overview of the data analysis steps and methods utilized in this study.

Figure 1 presents an overview of the data analyzed in this study and the analysis steps, the results of which are detailed below. Briefly, we compiled ChIP-seq (“Hughes”) or ChIP-exo (“Trono”) data for KZNF proteins from two different studies. Hughes data (60 KZNFs) is from Schmitges *et al.* 2016, while Trono data (221 KZNFs) is from Imbeault *et al.* (2017). We also identified motif data from other sources that correspond to each of the proteins in the combined list of 242 KZNFs (Matys *et al.* 2006; Encode-Project-Consortium 2012; Jolma *et al.* 2013; Kulakovskiy *et al.* 2013; Najafabadi *et al.* 2015b; Mathelier *et al.* 2016; Isakova *et al.* 2017; Yin *et al.* 2017). The Venn diagram in Supplemental Material, Figure S1 shows the overlaps among the data types.

For all analyses, we used two versions of the peaks from the Trono data. The first is the set of peaks reported in the original paper (Imbeault *et al.* 2017), and the second is the set of peaks obtained by rerunning the raw Trono reads using the same peak-calling system employed for the Hughes data. These “Trono reprocessed” peaks are, on average, 10-fold more numerous than the “Trono original” peaks, but they are only 1.8-fold less compared to those from the Hughes data, for the 39 KZNFs that overlap (average 40,660 for Hughes and 22,483 for Trono reprocessed).

For each KZNF, we computed the overlap in peaks (for the 39 present in both Hughes and Trono data), and the proportion of the Top 500 peaks (or maximum number of peaks where the number of peaks was <500) that overlap with each of 934 types of transposons and retroelements cataloged by the RepeatMasker track of the UCSC Table Browser (Karolchik *et al.* 2004). We also generated new motifs (using both MEME and RCADE) for both the Trono original and Trono reprocessed peak sets. As in our previous studies, we favored RCADE motifs, which are supported by the recognition code and therefore more likely to represent primary binding sites, but retained MEME motifs if RCADE produced no results [(a result of the algorithm failing to converge, or nonenrichment of any of the predicted motifs in the ChIP-seq peaks; see Najafabadi *et al.* (2015a)]. We also favored motifs derived from non-ERE peaks. We then scored the similarity among the motifs for each protein (choosing one per data set), and also scored the area under the receiver operating characteristic curve (AUROC) for all available motifs on the Hughes, the Trono original, and the Trono reprocessed data sets. The AUROC scores reflect the ability of the motif to discriminate peak sequences from the background (dinucleotide shuffled peaks). Finally, we selected a representative motif for each protein, favoring those with evidence for direct binding, and those with the highest AUROC on any ChIP data type. The web site accompanying this paper (http://kznfmotifs.ccbr.utoronto.ca/) contains files used in all the analyses herein, including the numerical data underlying the figures, as well as a visualization of the motifs and all the analyses for each protein considered. It also contains all the motifs as PWMs, and lists the representative motifs, together with confidence metrics for each: AUROC on ChIP data, similarity to most similar independently generated motif (if available), and method generated (RCADE, MEME, or external source).

### Overlap in peaks and EREs bound between Hughes and Trono data

**Figure 2:**
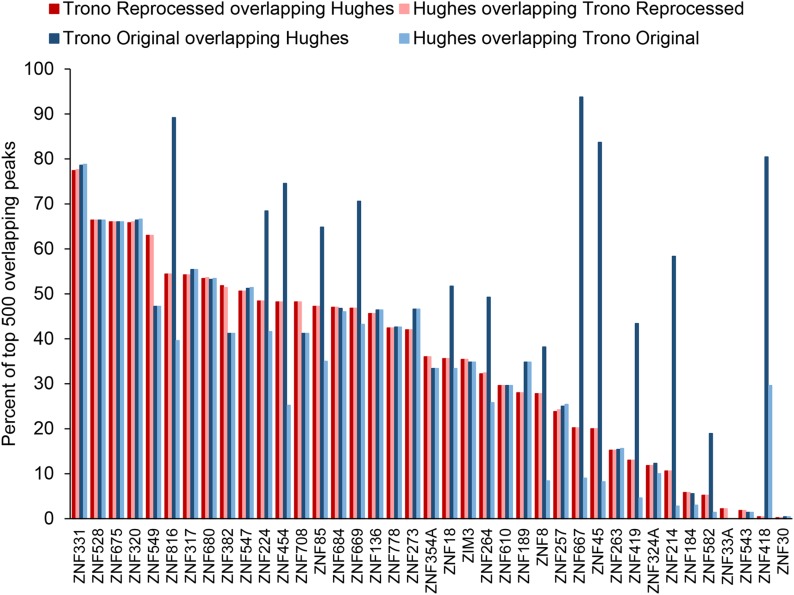
Peak overlaps for the 39 shared KZNFs between the Trono original and Hughes data (dark blue bars), Hughes data and Trono original data (light blue bars), Trono reprocessed and Hughes data (dark red bars), and Hughes and Trono reprocessed data (light red bars).

We first considered the overlap in peaks and the overlap in EREs bound between the Hughes and Trono data, for the 39 proteins that overlapped between the two studies. Figure 2 shows the percent peak overlap for each protein, calculated as the percentage of the top 500 Hughes peaks that overlap Trono peaks and vice versa in a range of ±250 bp from the peak summits, with the four bar graphs representing comparisons between Hughes data and Trono original and Trono reprocessed data. On average, 35% of the Top 500 Hughes and Trono reprocessed peaks overlapped in both comparisons, albeit with a considerable spread. At random, the overlap should be zero in all these comparisons, because the 500 peaks encompass a miniscule fraction of the genome. We observed no correlation between the degree of peak overlap and quality scores of the ChIP experiments (Kharchenko *et al.* 2008; Landt *et al.* 2012) (data not shown).

Our use of 500 peaks throughout is for convenience and uniformity; different numbers and proportions of peaks will yield slightly different numbers, but with generally similar conclusions (data not shown). For example, we note that the Trono original peaks often have a generally higher proportion of overlap with Hughes, relative to all other comparisons made. This phenomenon is explained by the data processing; there are a substantially lower number of peaks in the Trono original data (<500 in 17/39 KZNFs compared) and they are presumably the highest enriched based on the description in Imbeault *et al.* (2017). This trend is not evident with the Trono reprocessed data, and similar (albeit again not identical) outcomes were obtained with Trono original and Trono reprocessed data in the analyses below.

**Figure 3:**
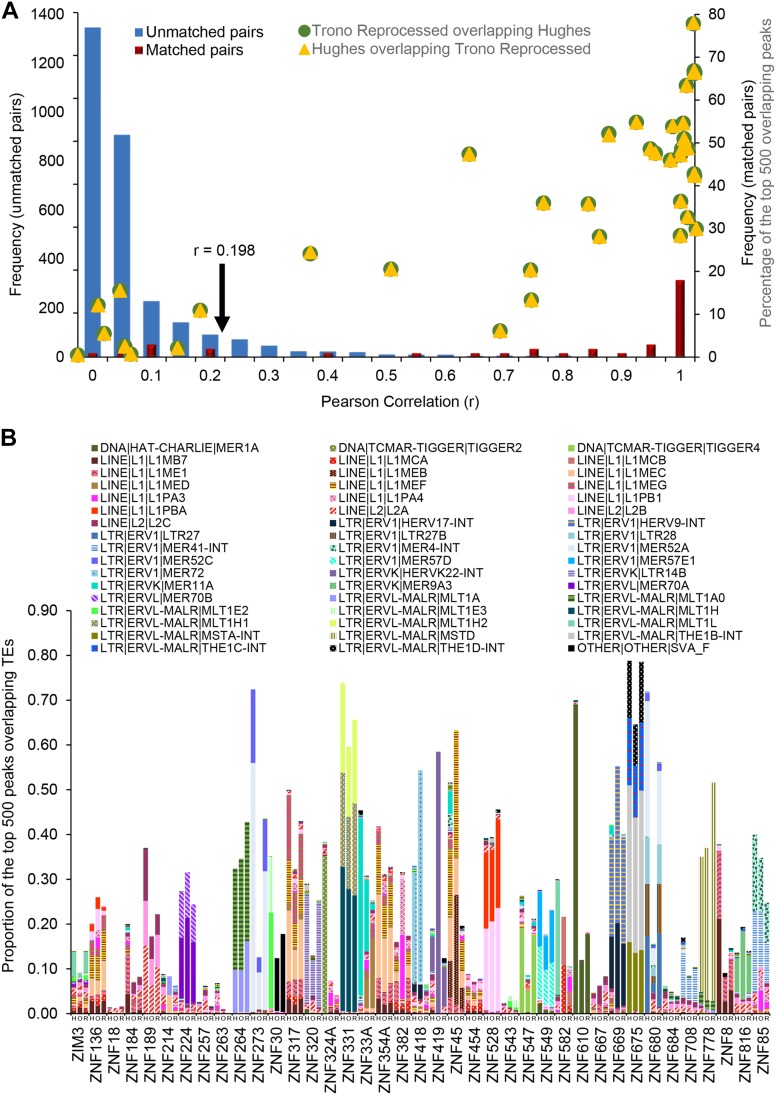
Overview of the ERE enrichment in Hughes and Trono ChIP data. (A) Pearson Correlation between the 39 Hughes and Trono reprocessed overlapping KZNFs (matched pairs; red bars) and nonoverlapping KZNFs (unmatched pairs: 2964 comparisons; blue bars) and the frequency of the KZNF pairs at each given correlation. The arrow indicates the correlation beyond which 82% of the matched pairs and 8% of the unmatched pairs lie. The percentage of the peak overlap between the Hughes and Trono reprocessed (yellow dots) and Trono reprocessed and Hughes (green dots) at corresponding correlations are also presented. (B) Fraction of the top 500 overlapping KZNFs enriched in TEs (ERE instances and transposons). In total, 51 single TE instances were enriched with a fraction of >0.1. H, Hughes; O, Trono Original; R, Trono Reprocessed.

Figure 3 provides a detailed view of the agreement in EREs bound in the two studies. Our comparison statistic is the Pearson correlation across all ERE classes, where the value for each is the proportion represented among the Top 500 peaks. Figure 3A shows that the distribution of values for the 39 matched KZNFs between the Hughes and Trono reprocessed data sets is much different from that for mismatched KZNFs; 82% (32/39) of the matched pairs exceed a correlation achieved by only 8% of the mismatched pairs. The distribution is quite similar when comparing Hughes and Trono original data, with 82% of the matched pairs exceed a correlation achieved by 5% of the mismatched pairs (data not shown). Figure 3B provides a visual confirmation that the individual transposons and EREs types represented in the three peak sets for each of the 39 proteins are largely in agreement.

Overall, we take this outcome to indicate that a large majority of data in both data sets correctly identifies the spectrum of EREs recognized, assuming that the overlapping KZNFs are an unbiased sample from each of the two studies. There can be good agreement on the EREs bound even when the peak overlap is relatively low; however, the higher peak overlap is usually associated with higher EREs correlation (Figure 3A). One interpretation of these observations is that both data sets are drawing from a substantially larger set of *bona fide* genomic binding sites, but both are subject to noise, and neither has been sequenced to saturation.

### Similarity of motifs from Hughes, Trono, and external data

We next compared the motifs obtained from the two ChIP data sets for the 39 overlapping proteins, as well as against the motifs obtained from other sources. For Hughes data, we used the motifs directly from the publication Schmitges *et al.* (2016). For the Trono data, we generated motifs for both the Trono original and Trono reprocessed peaks, using the same procedure employed in Schmitges *et al.* (2016). Briefly, we used either RCADE (Najafabadi *et al.* 2015a) or MEME (Bailey *et al.* 2009) to identify motifs based on the ±250 bp sequences around the summits of the top 500 peaks of each protein. We used motifs obtained by RCADE, using non-ERE peaks, if the algorithm was successful, since the fact that EREs within a given class are related by common descent can confound motif-finding algorithms. If non-ERE peaks did not generate significant motifs, we included the ERE peaks in the analysis. We used MEME to generate motifs if no RCADE motifs were obtained, selecting the top-scoring motif using CentriMo; similar to the RCADE motifs, priority was given to the motifs obtained by non-ERE peaks. Overall, 80% (48/60) of the motifs from the Hughes data, 74% (164/221) from the Trono original data, and 77% (170/221) from the Trono reprocessed were obtained from RCADE. RCADE was successful in at least one of the three data sets in the 39 shared proteins, and in many cases, on the same proteins from the Hughes and Trono laboratories (30/39 in common between Hughes, and at least one of the Trono original or Trono reprocessed data sets, 1/39 from Hughes only, 1/39 from Trono original only, and 7/39 from both Trono original and Trono reprocessed, but not Hughes).

**Figure 4:**
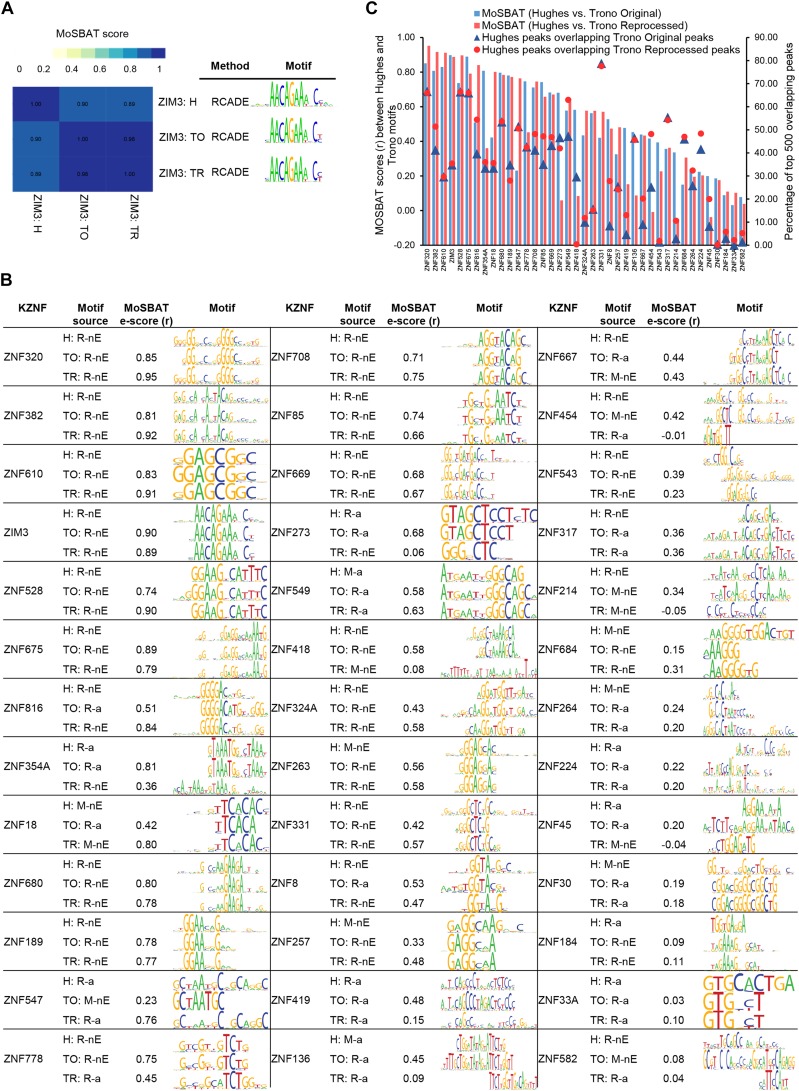
Similarity between ChIP-derived motifs. (A) Similarity between the Hughes and Trono motifs for ZIM3. The heat map on the left indicates the MoSBAT similarity e-scores between each pair compared. The motifs and the motif-finding methods are represented on the right. H, Hughes; TO, Trono Original; TR, Trono Reprocessed. (B) The MoSBAT e-scores between Hughes motifs and Trono original and Trono reprocessed motifs and the corresponding aligned motifs for the 39 overlapping KZNFs. H, Hughes; TO, Trono Original; TR, Trono Reprocessed; R, RCADE; M, MEME; a, all peaks; nE, nonERE peaks. (C) MoSBAT similarity e-scores for the 39 overlapping KZNFs between the Hughes data and Trono original (blue) and Trono reprocessed (red). The dots indicate the percentage of the overlap between the Hughes and Trono original peaks (blue) and Hughes and Trono reprocessed peaks (red).

To score similarity of motifs, we used MoSBAT (Lambert *et al.* 2016), which computes the Pearson correlation of the predicted affinities for two different motifs to thousands of randomly generated short sequences. MoSBAT is appropriate for this analysis because it is nonparametric, and requires no adjustments for differences in motif length. Figure 4A shows an example confirming similarity among multiple published motifs for ZIM3 (similar figures are shown for all KZNFs at http://kznfmotifs.ccbr.utoronto.ca/). In this example, only 35% of the top 500 Hughes peaks overlap Trono original and Trono reprocessed peaks, but RCADE produces very similar motifs with both data sets (*r* = 0.90 and *r* = 0.89, respectively). Figure 4B shows the motif similarity scores between the Hughes data and the Trono original and reprocessed data, and the corresponding motifs for the 39 overlapping KZNFs, illustrating that virtually all bear a clear visual resemblance, with only 3/39 (8%) and 7/39 (18%) of the KZNFs yielding similarity scores <0.10 between Hughes motifs and Trono original and Trono reprocessed motifs, respectively. Overall, roughly half of the overlapping KZNFs between the Hughes and Trono data (19/39; 49% for Trono Original and 18/39; 46% for Trono Reprocessed) yielded motif similarity scores >0.50, a value typically obtained from different experiments using the same transcription factor (TF) (Lambert *et al.* 2016). Furthermore, the majority of the overlapping KZNFs (33/39; 85% for Trono Original and 27/39; 69% for Trono Reprocessed) are clearly comparable by visual inspection (Figure 4B), and bear a similarity score of >0.20, which exceeds the score of the 98% of the mismatched KZNFs. As with the ERE overlap, in most cases the motifs tend to be similar, even when peak overlap between the two data sets is low. Higher peak overlap usually results in higher motif similarity, however (Figure 4C).

We also compared the ChIP-derived motifs with those from other sources, taken from the initial publications or TF databases. If there was more than one motif from the same source available for a KZNF, the one with the highest AUROC was selected for further analysis (41 in total; Matys *et al.* 2006; Encode-Project-Consortium 2012; Jolma *et al.* 2013; Kulakovskiy *et al.* 2013; Mathelier *et al.* 2016; Isakova *et al.* 2017; Yin *et al.* 2017), and 5/22 of Hughes KZNFs, 14/22 of Trono KZNFs, and 3/22 of both data sets overlapped with at least one external motif (Figure S1). For 23/41, the external in-vitro motif was similar (MoSBAT >0.2) to at least one ChIP-derived motif. Surprisingly, 17 of the external motifs outperformed the ChIP-derived motifs in predicting ChIP peaks (see the section *A reference motif set for human KZNFs* for details), among which six belonged to the SMiLE-seq motif sets (seven in total; Isakova *et al.* 2017), six to the HT-SELEX motif sets (12 in total; Jolma *et al.* 2013; Yin *et al.* 2017), and five to one of the other sources (10 in total; Matys *et al.* 2006; Encode-Project-Consortium 2012; Kulakovskiy *et al.* 2013). This outcome not only confirms that motifs derived *in vitro* are often consistent with *in vivo* genomic binding sites, but illustrates that the greater depth of the assays can produce more accurate motifs. Some cases, however, are discrepant (11/41 cases score <0.1 in MoSBAT compared to all corresponding ChIP-derived motifs), and will require resolution (see *Discussion*).

**Figure 5:**
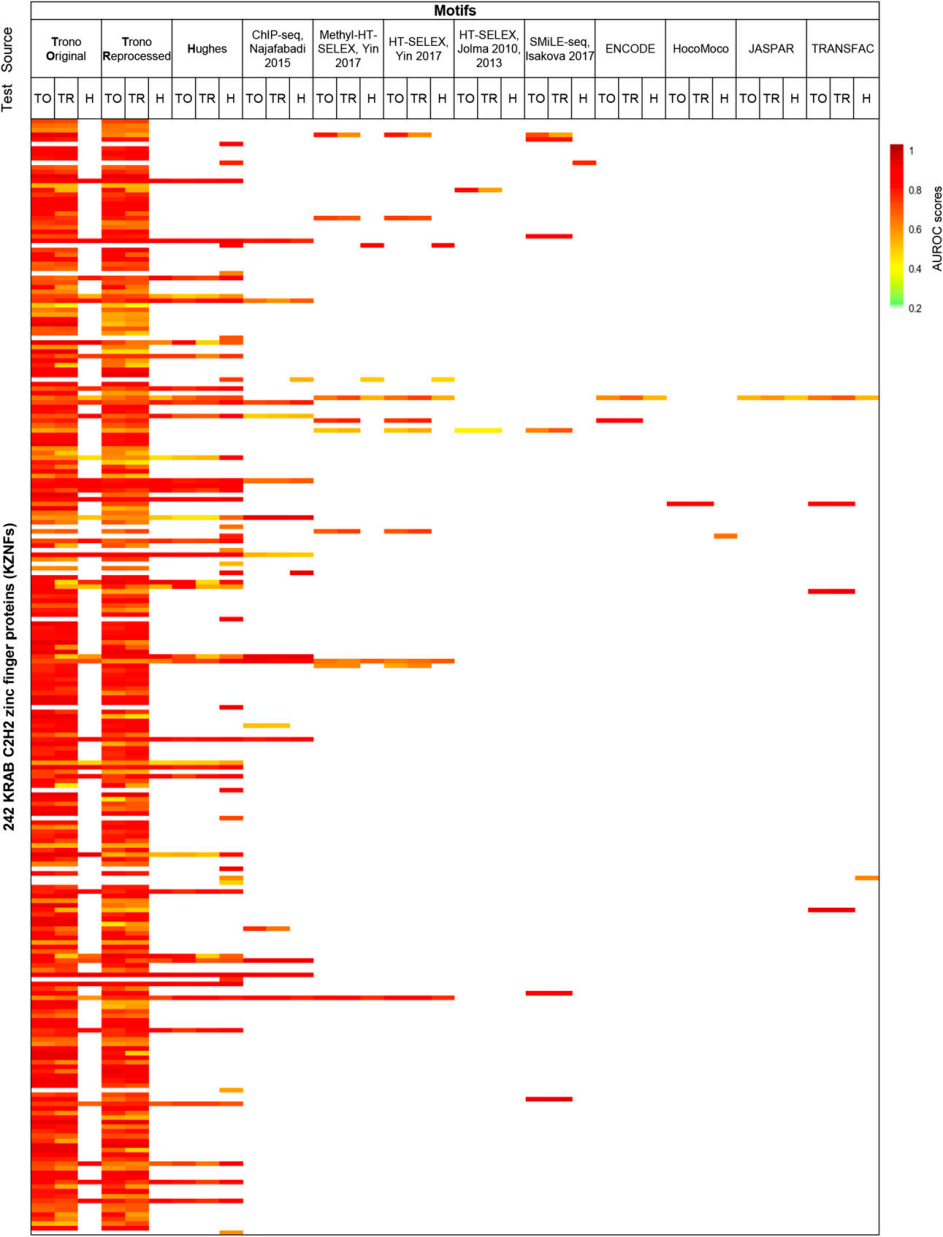
AUROC of the Hughes and Trono motifs and external motifs overlapping any of the two data sets. Heat map represents the AUROC value of each motif tested on Hughes, Trono original, or Trono reprocessed peaks. The first row at the top indicates the source of the motif, and the second row indicates the test data set. TO, Trono Original; TR, Trono Reprocessed; H, Hughes. White indicates no data is available. A full version of the figure that includes the KZNFs IDs is available at the web portal of the paper (http://kznfmotifs.ccbr.utoronto.ca/figures.html).

We also found that the vast majority of motifs obtained from one of the ChIP data sets were significantly enriched in peaks from the other. Figure 5 depicts AUROC values (Top 500 *vs.* dinucleotide shuffled sequences) for ChIP data and other sources tested on the three data sets: Hughes, Trono original, and Trono reprocessed. The left third of Figure 5 shows that, except for a few cases, the ChIP-derived motifs are comparable at predicting the ChIP peaks of the other data set, as well as the one from which they are derived. The right two-thirds of Figure 5 show that motifs from other sources are also generally good predictors of the ChIP peaks.

These analyses show that the two data sets typically identify similar binding motifs, most of which are also supported by the recognition code and/or by independent data.

### A reference motif set for human KZNFs

The comparisons above provide confidence metrics for the motifs obtained for each of the 242 KZNFs examined, representing reproducibility and predictive power both within and among data types, consistency with the zinc finger recognition code, and quality statistics for the data used to derive the motifs. We used these metrics to choose a single best current motif for each protein, as such a motif set is useful for many types of analyses (Matys *et al.* 2006; Encode-Project-Consortium 2012; Kulakovskiy *et al.* 2013; Mathelier *et al.* 2016).

The ranking system, intended to capture motifs that correspond to both ChIP data and external information, if available, is shown in Figure S2. We gave highest confidence to any motif (*in vitro* or ChIP-derived) that predicts “test” ChIP data (the data that they were not trained on: one of the Hughes, Trono original, or Trono reprocessed data sets) with AUROC at least 0.1 greater than all other motifs (Motif is uniquely predictive of test peaks = Class A). In the absence of a Class A motif, we then favored *in vitro* derived motifs that predict the test ChIP data better than, or almost as well as, the ChIP motifs (AUROC difference <0.1; *In vitro* motif predictive of test peaks = Class B), since *in vitro* data are not impacted by extraneous factors such as indirect binding. If no motifs satisfied these criteria, we selected ChIP motifs that are supported by the recognition code (*i.e.*, RCADE) (RCADE motifs = Class C), followed by the ChIP motifs that predict their training data better than all other motifs, where the AUROC was at least 0.1 greater than the others (ChIP motif is uniquely predictive of training peaks = Class D), followed by those that predict the test ChIP data sets even slightly better than the others (AUROC = 0.01–0.09 greater than the others) (Motif is most predictive of test peaks = Class E), and, finally, all remaining motifs including single motifs and motifs for three KZNFs (ZIM2, ZNF445 and ZNF785) that cannot be discriminated by any of the above criteria (Others = Class F).

**Figure 6:**
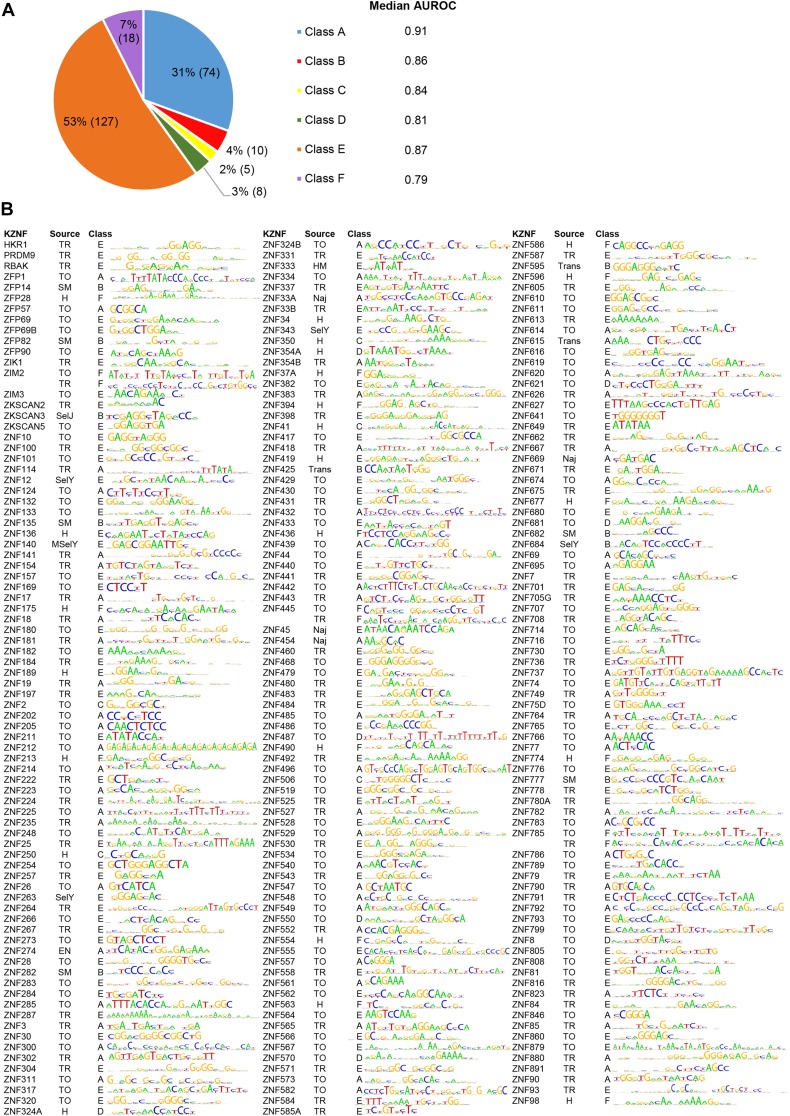
The reference motif set for the 242 KZNFs. (A) Percentage and number of the motifs (in parentheses) fit into classes A–F and the median AUROC values of each group. (B) The reference motif for each of the 242 KZNFs. Source refers to motif origin (TO, Trono Original; TR, Trono Reprocessed; H, Hughes; Naj, ChIP-seq (Najafabadi *et al.* 2015b); SM, SMiLE-seq (Isakova *et al.* 2017); SelY, HT-SELEX (Yin *et al.* 2017); MSelY, Methyl-HT-SELEX (Yin *et al.* 2017); SelJ, HT-SELEX (Jolma *et al.* 2013); EN, ENCODE; Trans, TRANSFAC; HM, HocoMoco). The class is the selection class that each motif falls into. For ZIM2, ZNF445 and ZNF785, both motifs from class F are represented.

Figure 6A shows the number and proportion of KZNFs currently falling into each class, and Figure 6B shows the motifs. In this scheme, the largest classes of motifs are Class A (in which one motif clearly outperforms all others on test data), and Class E (where all motifs are roughly equivalent). The median AUROC for each class does not vary greatly (Figure 6A). Most of the motifs are supported by the recognition code: 158/242 (65%) are RCADE motifs, 96% of which have AUC >0.6 predicting peak *vs.* nonpeak. As previously reported for KZNFs, these reference motifs are highly diverse, and tend to be longer and more information-rich than motifs for other TF types, which tend to be only 6–12 bases long (Badis *et al.* 2009; Jolma *et al.* 2013).

### Web portal for KZNFs

**Figure 7:**
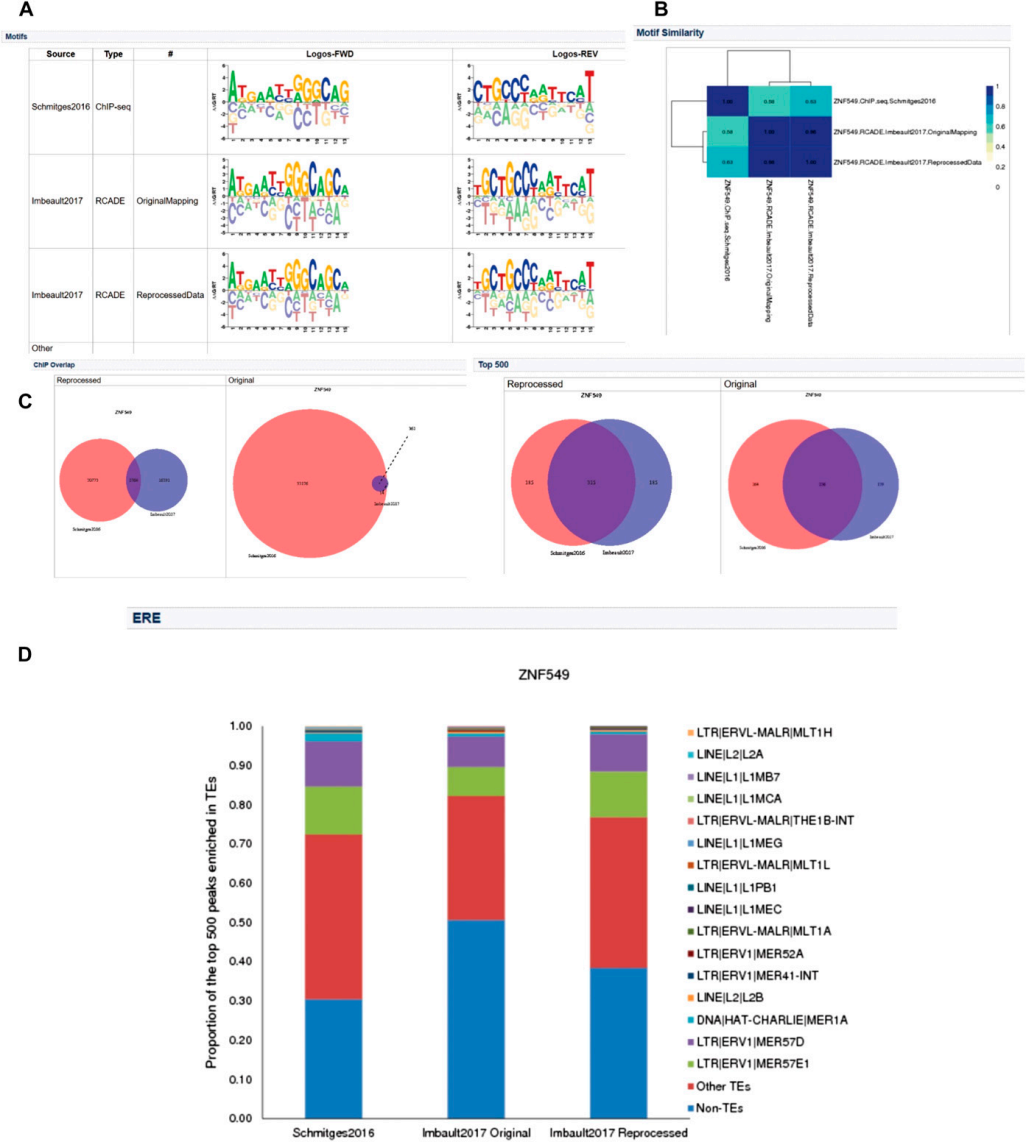
Web portal of ZNF549 containing all the analyses described (http://kznfmotifs.ccbr.utoronto.ca/report.php?name=ZNF549). (A) Motifs for the same KZNF derived from different sources. (B) MoSBAT similarity heat maps between all motifs. (C) Overlap between Hughes peaks and Trono reprocessed (left) and Trono original (right) peaks for all peaks and top 500 peaks. (D) ERE enrichment for the Hughes and Trono ChIP peaks.

Finally, we generated a web interface that assembles all the data described herein, providing all the relevant data files, as well as a graphical interface for each KZNF. Figure 7 provides an illustration for ZNF549, a KZNF with a Class A motif. Motifs from different sources are shown, if available (Figure 7A). A heatmap of the pairwise motif similarity scores between different motifs is then shown (Figure 7B). The peak overlap between the Hughes and Trono ChIP data are illustrated, where available, for all peaks and top 500 peaks (Figure 7C). Finally, the ERE enrichment of the peaks is given (Figure 7D).

## Discussion

The overall conclusion of these analyses is that there is reasonable agreement between the Hughes and Trono lab ChIP data, and also external data, on the motifs, genomic binding sites in HEK293 cells, and EREs enriched among those sites. This finding enables us to conclude that ChIP-seq—despite being a notoriously difficult method from which to derive motifs and to predict primary regulatory “targets”—is a viable strategy for both obtaining motifs and for identifying a potential physiological role for these proteins, or at least an initial driving evolutionary force (repression of EREs). These analyses also indicate that most human KZNFs are *bona fide* sequence-specific DNA-binding proteins.

Despite general overall agreement, there are discrepancies between the data sets. When considering both Trono original and Trono reprocessed data together, 23% of the experiments disagree on at least one of these parameters (EREs bound, *r* < 0.3 and motifs, *r* < 0.2); 3/39 (8%) of the experiments disagree on both. Because disagreement can stem from noise or error in only one of the two data sets, it is possible that these issues are less prevalent in the individual data sets (*e.g.*, 11.5% error in each of them would lead to an overall error of 23%). KZNFs for which the data sets disagree, and for which there are no motifs that relate to the recognition code (44/242), might be considered prime candidates for rerunning the ChIP assays, possibly in other cell types, and/or analysis by alternative assays such as SMILE-seq. Ideally, an *in vitro*-derived motif for each protein will eventually also be available, enabling confirmation that the motifs enriched in ChIP data represent *bona fide* direct sequence recognition events. In the meantime, the recognition code appears to provide a reasonable substitute.

The absence of a complete set of motifs for human TFs (Weirauch *et al.* 2014) remains a glaring shortcoming in the study of human gene regulation. Our collection of reference motifs includes 166 proteins for which there was previously no known motif. We anticipate that the reference set will be of general utility in the study of regulatory mechanisms and networks. It will also facilitate exploration of the targeting mechanisms of these proteins to EREs—the motifs alone are largely insufficient (Najafabadi *et al.* 2015b; Schmitges *et al.* 2016)—as well as their roles in human genetics and disease, because SNPs in noncoding regions often impact motif scores for TFs (Deplancke *et al.* 2016).

## Supplementary Material

Supplemental material is available online at www.g3journal.org/lookup/suppl/doi:10.1534/g3.117.300296/-/DC1.
