# Supplementary figures and images for: Comparison of ChIP-Seq Data and a Reference Motif Set for Human KRAB C2H2 Zinc Finger Proteins
Source: G3 (Bethesda). 2017 Nov 16;8(1):219–29. doi: 10.1534/g3.117.300296 (PMC5765350; doi:10.1534/g3.117.300296)

## Slide 1
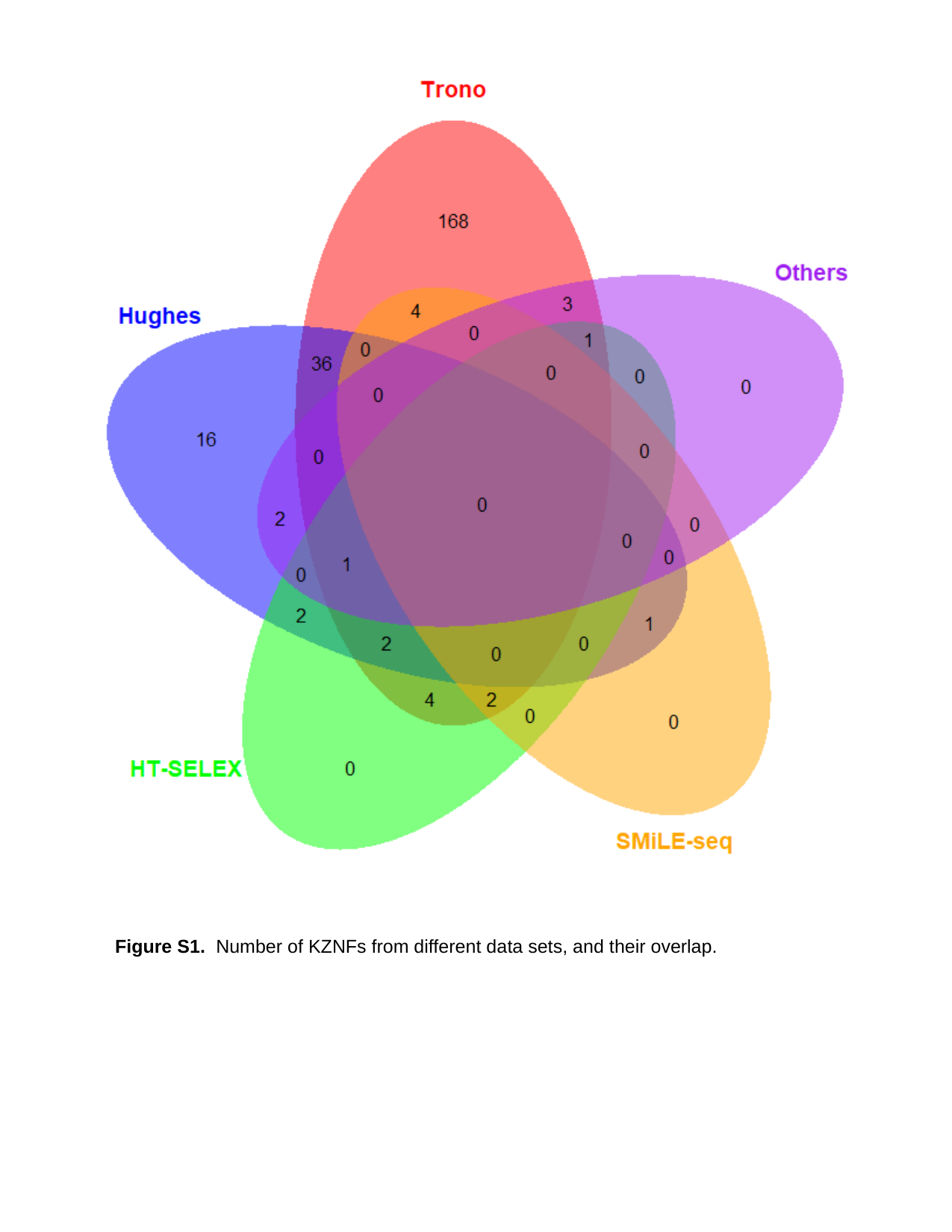

Figure S1. Number of KZNFs from different data sets, and their overlap.

Supplement: Supplementary file 1 [file 219FigureS1.pptx]
