# Supplementary material for: Comparison of ChIP-Seq Data and a Reference Motif Set for Human KRAB C2H2 Zinc Finger Proteins
Source: G3 (Bethesda). 2017 Nov 16;8(1):219–29. doi: 10.1534/g3.117.300296 (PMC5765350; doi:10.1534/g3.117.300296)
Supplement: Supplementary file 2 [file 219FigureS2.pptx]

## Slide 1
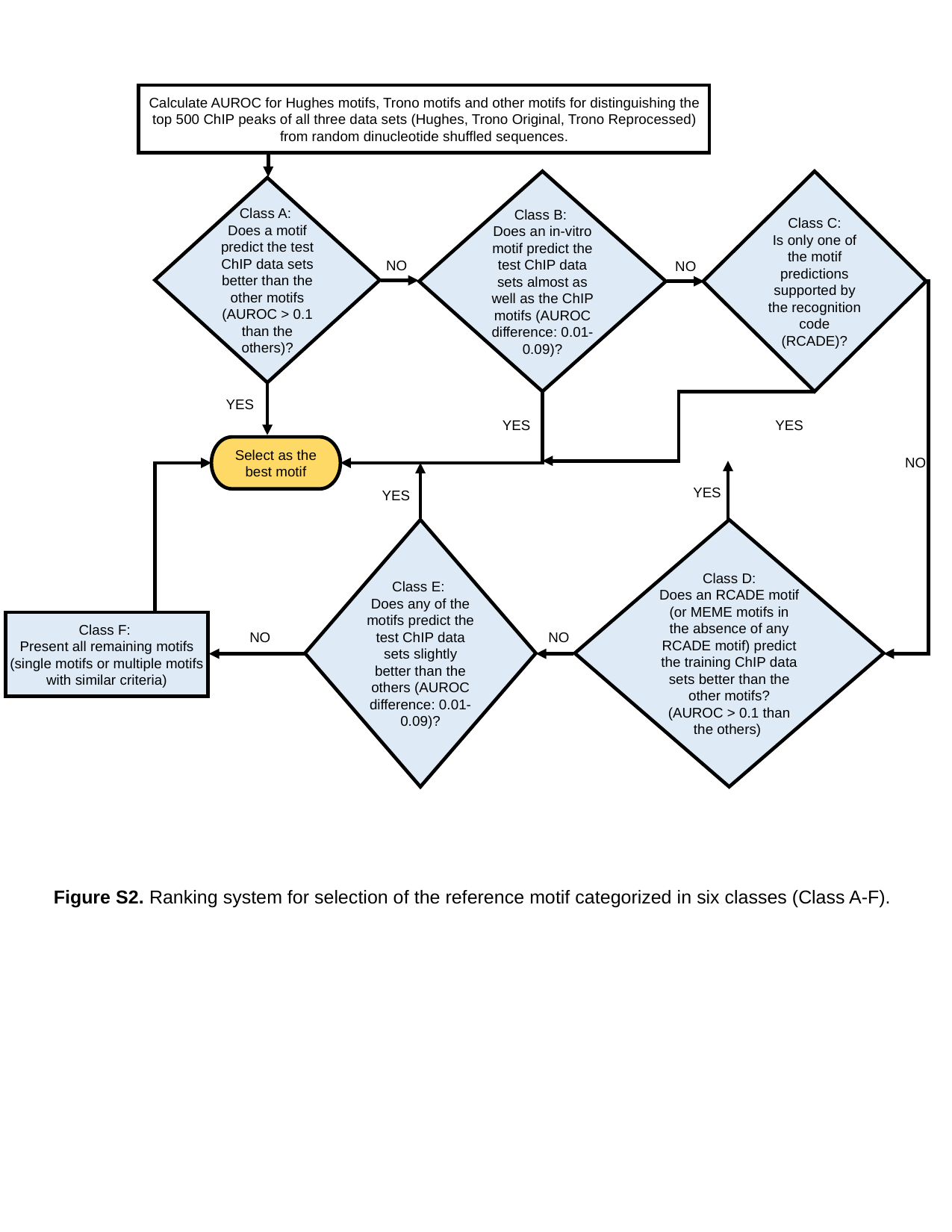

Calculate AUROC for Hughes motifs, Trono motifs and other motifs for distinguishing the top 500 ChIP peaks of all three data sets (Hughes, Trono Original, Trono Reprocessed) from random dinucleotide shuffled sequences.
Class B:
Does an in-vitro motif predict the test ChIP data sets almost as well as the ChIP motifs (AUROC difference: 0.01-0.09)?
Class C:
Is only one of the motif predictions supported by the recognition code (RCADE)?
Class A:
Does a motif predict the test ChIP data sets better than the other motifs (AUROC > 0.1 than the others)?
NO
NO
YES
YES
YES
Select as the best motif
NO
YES
YES
Class E:
Does any of the motifs predict the test ChIP data sets slightly better than the others (AUROC difference: 0.01-0.09)?
Class D:
Does an RCADE motif (or MEME motifs in the absence of any RCADE motif) predict the training ChIP data sets better than the other motifs? (AUROC > 0.1 than the others)
Class F:
Present all remaining motifs (single motifs or multiple motifs with similar criteria)
NO
NO
Figure S2. Ranking system for selection of the reference motif categorized in six classes (Class A-F).
